# Enhanced glucose forecasting using recurrent neural network and advanced feature engineering

**DOI:** 10.1038/s41598-026-41066-5

**Published:** 2026-04-09

**Authors:** Mahmoud H. Osman, Mennatullah Mahmoud, Salma Zakzouk, Samah Mohamed, Ibrahim Gomaa, M. Saeed Darweesh, Sayed Taha, Ahmed Soltan

**Affiliations:** 1 Acuanix Company, Cairo, Egypt; 2 Nanoelectronics Integrated Systems Center (NISC), Nile University, 26th of July Corridor, Sheikh Zayed City, 12588 Giza, Egypt; 3 Wireless Intelligent Network Center (WINC), Nile University, 26th of July Corridor, Sheikh Zayed City, 12588 Giza, Egypt; 4 Faculty of Computers and Artificial Intelligence, Cairo University, 12613 Cairo, Egypt; 5 Computers and Systems, Electrical Engineering Department, Fayoum University, 63514 Fayoum, Egypt

**Keywords:** Glucose Forecasting, Time Series Imputation, Recurrent Neural Network, Feature Engineering, Data processing, Machine learning

## Abstract

The aim of this study is to develop an artificial intelligence (AI)-driven pipeline for forecasting blood glucose levels to mitigate risks associated with hypoglycemia and hyperglycemia. The main research question focuses on the effectiveness of hybrid data preprocessing and feature engineering in enhancing glucose level predictions. The proposed approach employs a hybrid methodology for handling missing data and advanced feature engineering techniques. A recurrent neural network (RNN) model is developed to forecast glucose levels with a lead time of 30 minutes. The model is evaluated using metrics such as root mean square error (RMSE) and mean absolute error (MAE). Experimental results indicate that the proposed pipeline achieves an average RMSE of 19.64 ± 0.11 and an MAE of 13.54 ± 0.11 across all patients. The results demonstrate improved forecasting accuracy, enabling early detection of critical glucose fluctuations. The integration of hybrid preprocessing and RNN modeling effectively predicts glucose levels, providing valuable insights for diabetes management. This approach supports better prevention of glucose emergencies, ultimately enhancing the quality of life for individuals with diabetes.

## Introduction

The ”silent killer,” also known as diabetes mellitus, currently with unknown cure or even effective long-term treatment, presents serious health hazards and difficulties. The most critical aspect of diabetes is that individuals may be unaware of having the disease and may cause very severe long term health problems^1^. In 2019, it was anticipated that over 463 million individuals had diabetes, and by 2045, 700 million persons are expected to have the silent killer disease^2^.

Therefore, since the science did not discover a permanent cure for diabetes, it is critical to monitor and control blood glucose level (BGL) to prevent abnormal blood glucose activities, hypoglycemia and hyperglycemia. Hypoglycemia and hyperglycemia are mainly characterized by abnormal BGLs; which are, respectively, lower and higher than the normal level. Both hypoglycemia and hyperglycemia represents serious risks to diabetic patients. If hyperglycemia is poorly managed, it may cause long-term complications. On the other hand, hypoglycemia necessitates quick action to prevent potentially fatal conditions^3^.

Regular Continuous Glucose Monitoring (CGM), adherence to medication and insulin regimens, lifestyle changes, and prompt action are all necessary for maintaining good blood glucose control and minimizing the risks associated with hypoglycemia and hyperglycemia.

CGM devices and self-administered fingerstick tests are examples of traditional glucose monitoring techniques that have offered useful insights into glycemic management. However, these techniques frequently call for intrusive procedures, might be uncomfortable for people, and might not offer real-time data^4^.

Systems for monitoring and predicting blood glucose levels with AI have various benefits. First off, they can enable continuous real-time glucose monitoring, doing away with the need for frequent fingerstick tests and providing a more thorough knowledge of glucose dynamics^5^. Second, AI can see patterns, trends, and possible risk factors that medical personnel might miss, allowing for early intervention and customized treatment plans^6^.

**Fig. 1:**
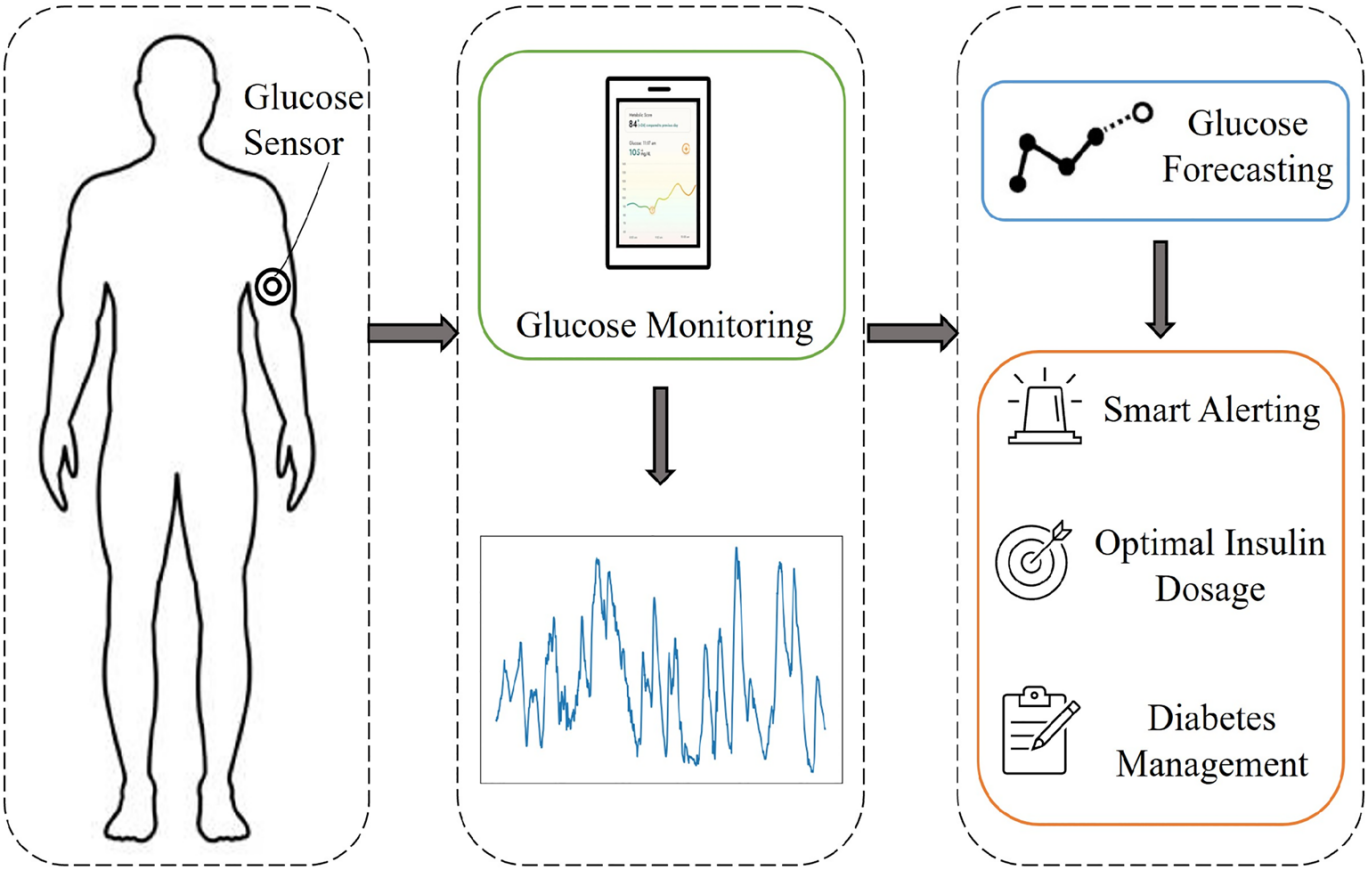
Whole system illustration starting from glucose sensing, to benefits of glucose forecasting.

Designing a BGL forecasting algorithm that can accurately anticipate glucose levels with a lead time of 30 minutes is the goal of this study. The study seeks to aid in avoiding hypoglycemia and hyperglycemia episodes, so improving disease management and quality of life for people with diabetes as summarised in Fig. 1.

The main contributions of this study are as follows:

- Introduces derived features that capture additional patterns beyond raw data.
- Demonstrates improved forecasting accuracy, surpassing existing literature on blood glucose level prediction.
- Encodes contextual information to predict subtle changes in glucose levels effectively.

After conducting a thorough literature review in Section Literature review, this paper is divided into four other main sections. Section Methodology illustrates the methodology, which discuss Data preprocessing, and modeling. Moreover, the results of the study are then discussed in Section Results & discussion, and finally, Section Conclusion presents the conclusion. To improve clarity and assist readers, Table 1 provides a list of abbreviations used throughout the manuscript.

**Table 1:** Abbreviations used in this wtudy.

Abbreviation	Definition
CGM	Continuous Glucose Monitoring
BGL	Blood Glucose Level
T1D	Type 1 Diabetes
RNN	Recurrent Neural Network
LSTM	Long Short-Term Memory
CNN	Convolutional Neural Network
MAE	Mean Absolute Error
MSE	Mean Squared Error
RMSE	Root Mean Squared Error
MLP	multi-layer perceptron
MPC	model predictive control
SEG	surveillance error grid
GRU	Gated Recurrent Unit
GANs	Generative Adversarial Networks
VAE	Variational Autoencoder
ARIMA	AutoRegressive Integrated Moving Average
CEG	Clarke Error Grid

## Literature review

**Fig. 2:**
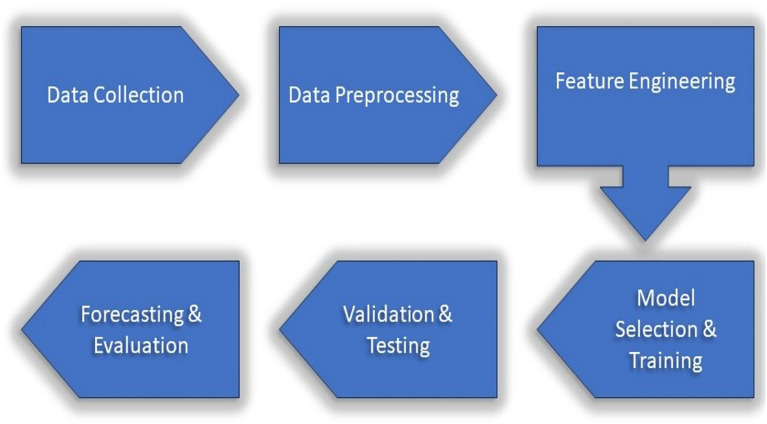
Pipeline steps of time series forecasting.

The time series forecasting pipeline typically involves several steps, as shown in Fig. 2. It begins with data collection, where historical records, such as blood glucose measurements, are obtained. This is followed by data preprocessing, which includes handling missing values, treating outliers, and converting data into a format suitable for modeling. Feature engineering is then performed to extract meaningful features that enhance forecasting accuracy. The final stages involve model selection, training, validation, testing, prediction, and evaluation of the forecasting model.

BGL prediction models are generally classified into three categories: physiological models that require extensive domain knowledge, hybrid models that use intermediate knowledge, and data-driven models that rely solely on data and adopt a black-box approach^7^. Data-driven models are commonly employed for linking the past, present, and future values of BGL, often utilizing machine learning and time-series techniques. This literature review is divided into two subsections: preprocessing methods and prediction models. Preprocessing plays a critical role in improving the performance of forecasting models by preparing the data for analysis. For handling missing data^8^, applied linear interpolation for training datasets and linear extrapolation for testing datasets.

In another approach^9^, addressed gaps in training datasets using a first-order linear model for up to three consecutive missing samples. For longer gaps, a statistical model based on glucose dynamics was applied. In testing datasets, prior CGM measurements were used to fill gaps until new observations became available. Similarly^10^, replaced missing CGM data with estimates from earlier phases of processing. The authors of^11^ compared raw data, linear interpolation, and a time-series interpolation technique, finding that time-series interpolation provided the highest accuracy, followed by linear interpolation.

Various approaches have been proposed to forecast BGL using preprocessed data. For example^12^, developed a prediction model based on a multi-layer perceptron (MLP) using statistical features and recent BGL values as inputs. This approach demonstrated strong performance in terms of RMSE and other evaluation metrics across different prediction horizons. Similarly^10^, introduced a model predictive control (MPC) framework with adaptive control rules that incorporated insights from CGM data and insulin doses to manage BG concentrations effectively. Recurrent neural networks (RNNs), particularly Long Short-Term Memory (LSTM) networks, were explored in^13^ to predict BGL up to one hour in advance. These models were trained end-to-end with past BGL data and evaluated using metrics such as RMSE and the surveillance error grid (SEG). Deep learning models were further explored in^14^, where LSTM, WaveNet, and Gated Recurrent Unit (GRU) networks were combined using weighted decision-level fusion. This approach showed improved accuracy across multiple prediction horizons. Finally^8^, examined ensemble techniques, including stacking, multivariate, and sequencing approaches. Among these, stacking demonstrated superior performance, achieving the best results in terms of RMSE, MAE, MCC, and SEG metrics.

While other advanced methodologies have been introduced, such as Transformer-based models. For instance, Informer and Transformer-XL leverage self-attention mechanisms to model global temporal dependencies efficiently, making them suitable for multivariate and high-frequency CGM data. Although Informer introduces a ProbSparse attention mechanism to reduce computational complexity, it still suffers from being a highly complex methodology compared to RNN-based models^15,16^. Moreover, the field has seen the integration of generative models such as Variational Autoencoders (VAEs) and Generative Adversarial Networks (GANs) to enhance the robustness of forecasting by generating synthetic patient profiles, augmenting training data, and estimating uncertainty. For example, recent work explored GANs to synthetically generate CGM-like time series, significantly improving the performance of downstream predictors^17^.

Although this study focuses solely on CGM data—since additional features such as food intake are often unavailable or unreliable in real-world scenarios—there have been significant advancements in AI-driven data acquisition, such as automated food recognition, that demonstrate the potential for enriching diabetes management systems. For instance, deep learning-based food classification models have been proposed to support dietary logging and nutritional tracking, which can directly impact blood glucose regulation. Notable works include a MobileNetV2-based architecture for accurate, real-time food recognition in the food industry^18^, and another that leverages EfficientNetB7 combined with CBAM attention and transfer learning for robust food classification under variable conditions^19^.

Table 2 provides a summary of the related work, datasets, performance metrics, prediction horizons and their limitations, organized chronologically.

**Table 2:** Literature review wummarized results.

Work	Dataset	Input Features	Model	Window (min)	Metric	Results (mg/dl)	Limitations
^9^	OhioT1DM	CGM, IOB	LSTM	30	RMSE	19.37	Uses insulin which is often unavailable
^13^	OhioT1DM	CGM	RNN	30,60,120,180	RMSE	18.87±1.79	Ignores missing data; no hybrid preprocessing
					SE	0.186±0.047	
^12^	DirecNet^20^	CGM	ANN	30	RMSE	6.31	Small dataset; no hybrid methods
^21^	OhioT1DM	CGM, IOB, Meal	Dilated RNN	30,60,90	RMSE	18.9	Complex model; no CGM-only focus
^14^	OhioT1DM	CGM	Hybrid (LSTM, GRU, etc.)	30	RMSE	21.90	Complex model; no CGM-only focus
^22^	DirecNet^20^	CGM	FEEMD	30	MAE	5.65	limited dataset validation
					RMSE	6.92	
					MAPE	6.12%	-
^8^	OhioT1DM	CGM	Ensemble (LR, LSTM, BiLSTM)	30,60,90,120	RMSE	19.63	Higher model complexity
					MAE	13.88	
					MCC	0.756	-
					SEG	0.204	-
^11^	OhioT1DM	All params	LSTM	-	RMSE	14.24	Uses all features
					MAE	14.08	
^10^	Not Mentioned	CGM, carbs, insulin	-	-	RMSE	9.93±2.02	No dataset info; no hybrid focus
This Methodology	OhioT1DM	CGM	RNN	60	RMSE	19.24±2.07	
					MAE	13.64±1.40	

## Methodology

**Fig. 3:**
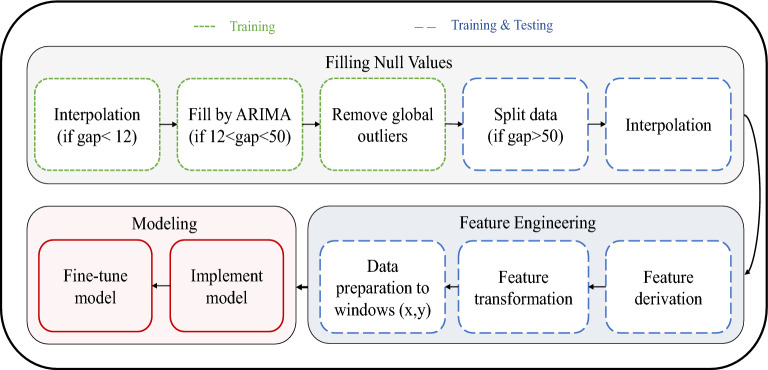
The proposed pipeline for smart blood glucose level prediction.

The aim of this paper is to develop a Smart BGL framework to forecasting the BGLs of diabetic patients. The proposed methodology follows a structured pipeline as outlined in Fig. 3. It begins with selecting and exploring the CGM dataset, followed by tailored preprocessing to handle missing values based on gap lengths. Afterward, domain-specific features are engineered and transformed to enhance model learning. These processed features are then fed into a RNN model optimized for short-term forecasting.

### Dataset exploration

Upon reviewing the available literature for datasets, it is discovered that the Ohio dataset is widely adopted for benchmarking. Twelve people with type 1 diabetes provided data for eight weeks, which are included in the OhioT1DM Dataset^23,24^. The dataset includes a wide range of data, including BGLs from CGMs measured every five minutes, BGLs from sporadic finger-stick self-monitoring, insulin doses (bolus and basal), self-reported mealtimes with carbohydrate estimates, and self-reported times for exercise, sleep, work, stress, and illness.

A single feature, the glucose level, is chosen as a CGM data point, collected at five-minute intervals, for each record in the collection, which consists of 19 data fields overall.

Although the dataset is relatively small, containing only 12 subjects, it still offers notable diversity. It includes balanced gender representation (6 male, 6 female), a broad age range (20–80 years), and two cohorts collected in different releases: the 2018 version with Basis Peak bands and the 2020 version with Empatica Embrace bands. These characteristics make the OhioT1DM dataset a valuable benchmark, despite its modest size, and explain its frequent adoption in the literature for blood glucose prediction research.

### Data pre-processing

#### Handling null values

**Fig. 4:**
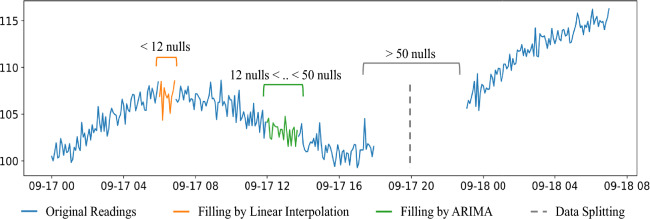
Hybrid method of filling gaps according to the gap length.

In accordance with the length of missing values within the dataset, a specific methodology for imputing these null values is selected. This imputation strategy is guided by thresholds. These threshold values are essentially cutoff points that help guide the decision-making process for selecting the most appropriate imputation technique based on the length or extent of the missing data within a dataset. Fig. 4 visualizes this hybrid technique; it consists of three thresholds.

1. Threshold 1: Less than 12 Consecutive Null Values
  When the number of consecutive null values in a dataset is fewer than 12, it is considered a relatively small gap in the data.
  In this case, an interpolation method is employed to fill the missing values. Interpolation techniques estimate missing values based on the patterns and values of adjacent data points. Spline interpolation is used.
2. Threshold 2: Between 12 and 50 Consecutive Null Values
  When the consecutive null values fall within the range of 12 to 50, it signifies a moderately sized gap in the data.
  To handle such cases, an AutoRegressive Integrated Moving Average (ARIMA) model is applied. ARIMA models are time-series forecasting models that can capture underlying trends and seasonality in data, making them suitable for imputing missing values within this range. After forecasting consecutive null values, there is a possibility that outlier values may appear. In such cases, it becomes necessary to eliminate these outliers while disregarding any global outliers. Subsequently, the count of null values is assessed; if it falls below 12, a second round of interpolation is applied. Then, if the count exceeds 12, the data is partitioned into distinct dataframes.
3. Threshold 3: Greater than 50 Consecutive Null Values
  If the number of consecutive null values exceeds 50, it suggests a substantial gap in the data, which requires a specialized approach. In such instances, the method employed is data cutting. This approach is designed to handle large gaps or missing data patterns that may not be effectively addressed by standard techniques like interpolation or ARIMA.

#### Feature engineering

The goal is to forecast glucose levels with the previously selected CGM single feature. However, to enhance prediction accuracy, several derived features are introduced. These features are presented in Table 3.

**Table 3:** Features derived from glucose readings with their description.

Feature Derived	Description
diff1	Representing the difference between the current glucose reading and the preceding one
diff6	Indicating the difference between the current glucose reading and the reading recorded six instances prior.
trend	Reflecting the trend of glucose readings over the past hour.
avg	Denoting the average glucose level observed in the previous hour.
std	Signifying the standard deviation of glucose readings within the last hour.
day part	Splitting Day into 7 parts based on the hour of the day.
date—dayOfWeek	Representing which day of the week ranging from 1 to 7.
date—hourOFDay	Representing which hour of the day ranging from 1 to 24.

**Fig. 5:**
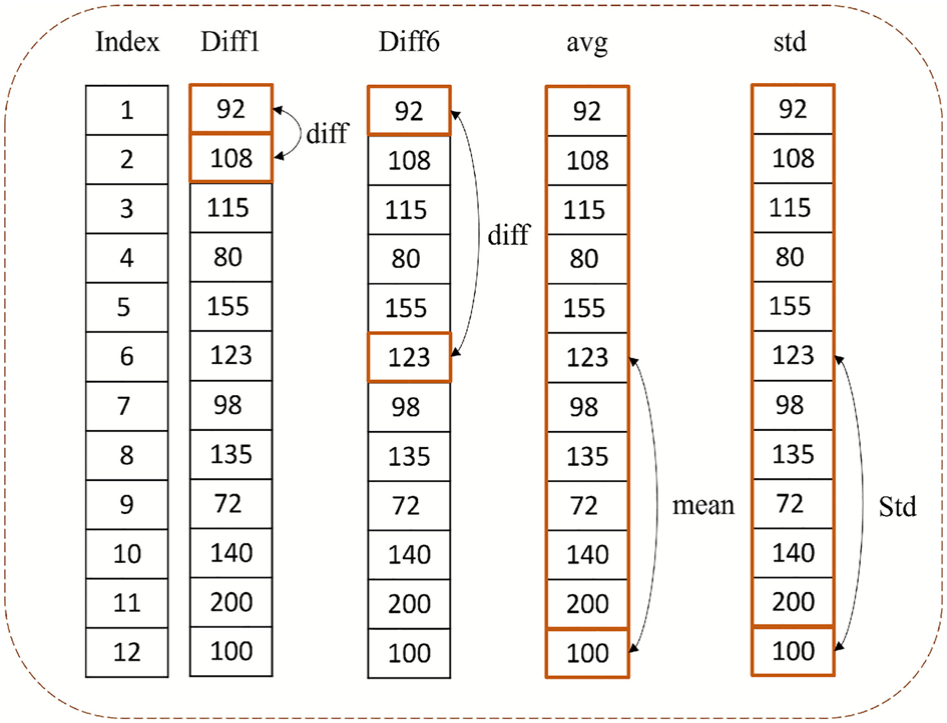
Illustration of derived features from glucose readings.

Additionally, the two features linked to the recording date—dayOfWeek and hourOFDay are integrated to provide temporal context for the analysis. These features collectively enhance the dataset and facilitate more accurate glucose level forecasting. An example visualization of these features is shown in Fig. 5.

To assess their relevance to hypoglycemia and hyperglycemia detection, an analysis is conducted where each glucose reading was labeled into one of three classes: hypoglycemia (<70 mg/dL), normal (70–180 mg/dL), and hyperglycemia (>180 mg/dL). A decision tree classifier is employed to estimate feature importance based on classification relevance to these abnormal glycemic states. The results presented in table 4 reveal that the most strongly correlated feature with glycemic states was avg (correlation: 0.709), followed by trend (0.445), day part (0.357), diff6 (0.357), and std (0.324). Time-based features such as hour of day and day of week also demonstrate moderate correlation, highlighting the circadian influence on glucose levels.

**Table 4:** Correlation of derived features with hypoglycemia and hyperglycemia labels.

Feature	Correlation with Label
avg	0.709
trend	0.445
day_part	0.357
diff6	0.357
std	0.324
hour_of_day	0.300
day_of_week	0.276
diff1	0.242

Although the OhioT1DM dataset includes additional inputs such as insulin dosage and meal intake, only CGM readings are utilized in this study to prioritize real-world applicability. In daily settings, supplementary data such as insulin and carbohydrate entries are often missing, inconsistent, or manually reported, leading to potential noise and reduced model robustness. In contrast, CGM data is reliably and continuously recorded, making it the most dependable source for practical deployment. Therefore, the feature engineering process are deliberately restricted to CGM-derived features to ensure generalizability and feasibility in typical usage scenarios.

#### Feature transformation

This step refers to the process of converting the existing features in a dataset to create new representations that are more informative or suitable for a particular data analysis or machine learning task. There are various techniques for feature transformation, in this study, log transformation is employed to solve the problem of data skewing—to make all column values in the same range. As mentioned in the literature^25^, using discriminative feature transformation for sensor data is still a very uncommon technique, however, this cutting-edge method is adopted.

### Pipeline for testing data

By eliminating unnecessary procedures that are already carried out during training, a specific pipeline is executed to compare the performance of the proposed model against models developed using the same ”Ohio” dataset:

1. Interpolate and predict nulls without threshold
2. Don’t cut data frame
3. Calculate The derived features and transform the data and the values that are not null in the original file. In production, last four steps in the training pipeline is calculated.

### Modeling

This section outlines the chosen model type, providing detailed description of the chosen architecture, including layers and finely tuned hyperparameters, followed by the evaluation criteria implemented to assess the performance of this model.

#### Model architecture & hyperparameters

Following experimentation with various models including simple RNN and LSTM networks, it was observed that simple RNNs performed better. This suggests the presence of short-term dependencies within the data and the absence of a necessity to capture long-range dependencies. This implies that RNN suffices for addressing the problem without the need for introducing unnecessary complexity by using a more complex model.

This simple architecture is chosen due to its balance between temporal modeling capability and computational simplicity. Unlike deep or complex architectures such as Transformers, the RNN is lightweight and well-suited for capturing short-term temporal dependencies in glucose data. This choice aligns with the deployment objective, as the model is intended for real-time use where a lower-cost model will be more efficient. Furthermore, the pipeline includes extensive feature engineering and domain-specific preprocessing that encapsulate many of the temporal and contextual patterns in the data. This reduces the need for the model to extract such patterns internally, diminishing the marginal benefit of using deeper or attention-based architectures.

**Fig. 6:**
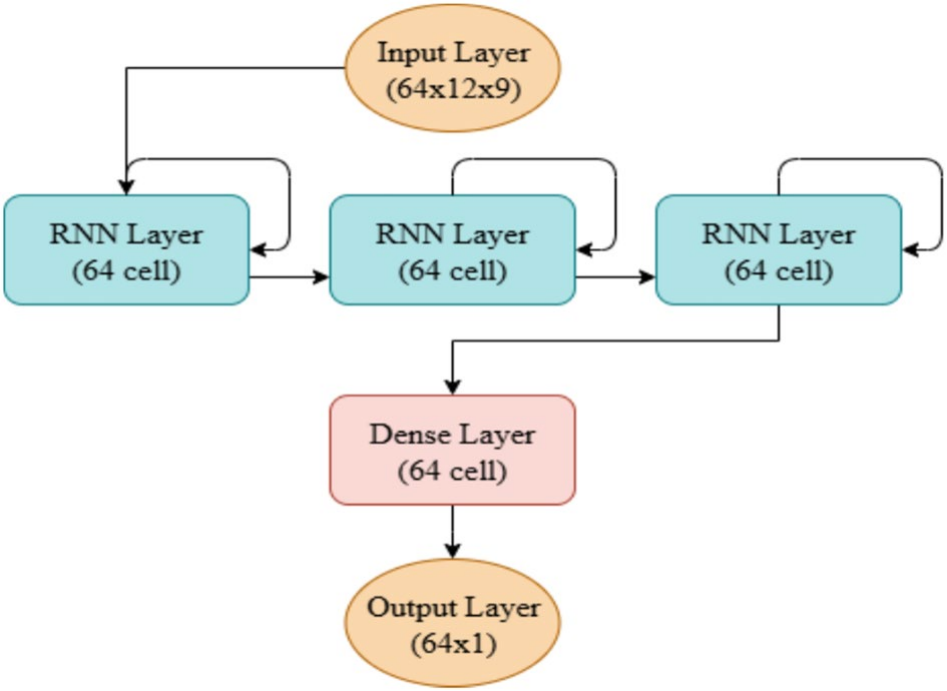
Proposed model architecture.

The exact model architecture and hyperparemeters used are presented in Table 5 and visualized in Fig. 6. The model consists of 3 RNN layers of followed by 2 linear layers, all with 64 neurons. Adam optimizer was employed with learning rate set at 1e-3. Additionally, training data was processed in batches, each with a size of 64.

**Table 5:** RNN hyperparameters.

Parameter	Value
Length of sequences	12
NUM Input channels	9
Batch size	64
NUM RNN layers	3
NUM linear layers	2
Hidden size	64
Learning rate	.001

In this study, the prediction horizon is set to 30 minutes, which is equivalent to 6 readings. This is chosen based on both clinical intervention needs and established literature ^26^. Importantly, it is demonstrated that shorter horizons (e.g., 15 minutes) limit the capacity for insulin or carbohydrate interventions due to physiological delays, while longer horizons introduce greater predictive uncertainty ^26^. Similarly, 15- and 60-minute horizons were compared and reported that the 30-minute range provides a clinically safe window for preventive measures, with high accuracy captured in SEGs (>99% safe zone at 15 minutes, 95% at 60 minutes) ^27^. From a medical standpoint, fast-acting insulin requires approximately 15–30 minutes to begin lowering glucose levels, while carbohydrate intake can take up to 20 minutes to correct hypoglycemia ^28^. Thus, the 30-minute window ensures adequate lead time to administer fast-acting insulin or carbohydrates and maintain predictive reliability. Additionally, a history window size of 12 readings is chosen, corresponding to 60 minutes.

## Results & discussion

The performance of the model is evaluated on those 12 patients according to the previously mentioned evaluation metrics.

### Model evaluation

**Fig. 7:**
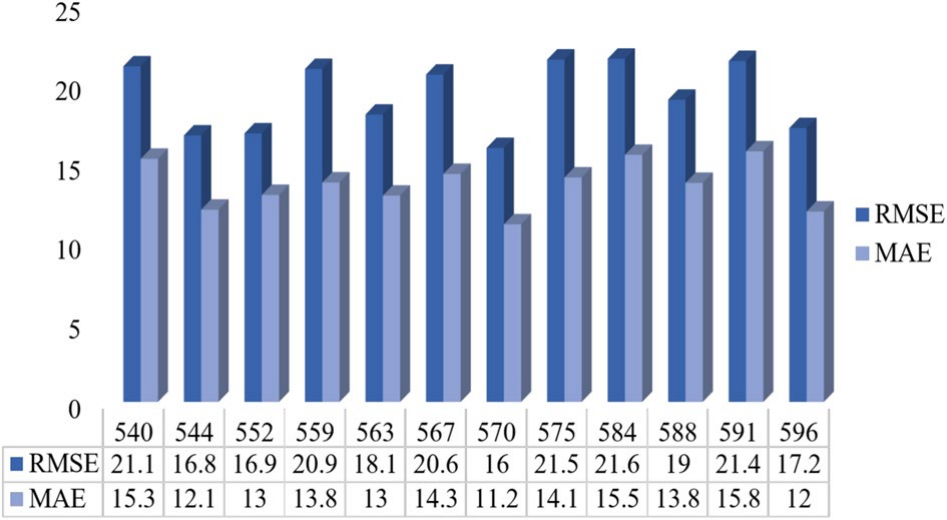
RMSE & MAE of the proposed model for all patients.

Table 6 presents all results of each patient’s model. The errors of all 12 models are visualized in Fig. 7 to ease their comparison.

**Table 6:** Results of the proposed model with additional metrics.

Patient ID	RMSEs	MAEs	R$^{2}$	MCC
540	$21.09 \pm 0.11$	$15.28 \pm 0.08$	$0.902 \pm 0.014$	$0.794 \pm 0.018$
544	$16.75 \pm 0.06$	$12.08 \pm 0.07$	$0.939 \pm 0.012$	$0.855 \pm 0.017$
552	$16.88 \pm 0.17$	$13.01 \pm 0.29$	$0.942 \pm 0.016$	$0.813 \pm 0.015$
559	$20.93 \pm 0.09$	$13.79 \pm 0.06$	$0.935 \pm 0.013$	$0.846 \pm 0.019$
563	$18.06 \pm 0.03$	$12.97 \pm 0.09$	$0.860 \pm 0.015$	$0.803 \pm 0.020$
567	$20.57 \pm 0.11$	$14.33 \pm 0.13$	$0.838 \pm 0.017$	$0.768 \pm 0.018$
570	$15.96 \pm 0.08$	$11.16 \pm 0.12$	$0.978 \pm 0.010$	$0.903 \pm 0.014$
575	$21.52 \pm 0.45$	$14.11 \pm 0.16$	$0.878 \pm 0.018$	$0.813 \pm 0.021$
584	$21.57 \pm 0.11$	$15.53 \pm 0.12$	$0.849 \pm 0.015$	$0.794 \pm 0.019$
588	$18.99 \pm 0.06$	$13.77 \pm 0.02$	$0.878 \pm 0.012$	$0.809 \pm 0.017$
591	$21.43 \pm 0.12$	$15.75 \pm 0.12$	$0.814 \pm 0.019$	$0.679 \pm 0.022$
596	$17.21 \pm 0.07$	$11.97 \pm 0.04$	$0.915 \pm 0.011$	$0.794 \pm 0.015$
Average	$19.24 \pm 2.07$	$13.64 \pm 1.40$	$0.894 \pm 0.044$	$0.806 \pm 0.049$
Median	20.25	13.89	0.890	0.806
95% CI	[19.61, 24.15]	[13.98, 16.59]	[0.863, 0.925]	[0.772, 0.840]

The model surpasses the findings reported in the Literature Review section by demonstrating superior proficiency in forecasting glucose levels. The RMSE values ranged between 15.96 and 21.57 with margins of error between 0.03 and 0.45. Among all patients, ID 570 achieved the lowest error (15.96), while ID 584 recorded the highest (21.57), reflecting variability in individual glucose dynamics. On average, the model achieved an RMSE of $19.24 \pm 2.07$ and an MAE of $13.64 \pm 1.40$. When considering confidence intervals across patients, RMSE was 21.88 (95% CI: 19.61–24.15) and MAE was 15.29 (95% CI: 13.98–16.59), confirming the stability of performance estimates. Beyond error-based metrics, the model attained an average R$^{2}$ of $0.894 \pm 0.044$ (95% CI: 0.863–0.925), showing that nearly 90% of the variance in glucose trajectories was explained by the predictions. Similarly, the average MCC was $0.806 \pm 0.049$ (95% CI: 0.772–0.840), demonstrating balanced classification performance across hypoglycemia, normoglycemia, and hyperglycemia. These findings confirm not only high predictive accuracy but also robustness and consistency across patients, reinforcing the clinical reliability of the proposed framework.

Although fitting an individual model to each patient can be computationally expensive, it allowed personalized forecasting. This approach takes each the unique pattern of glucose readings into consideration which leads to tailored predictions for individual patients. Besides, RNN architecture is found to be the proper model for the problem that forecasting glucose level forecasting does not require long historical data, and one hour is considered an acceptable window and commonly used in literature^29^.

**Fig. 8:**
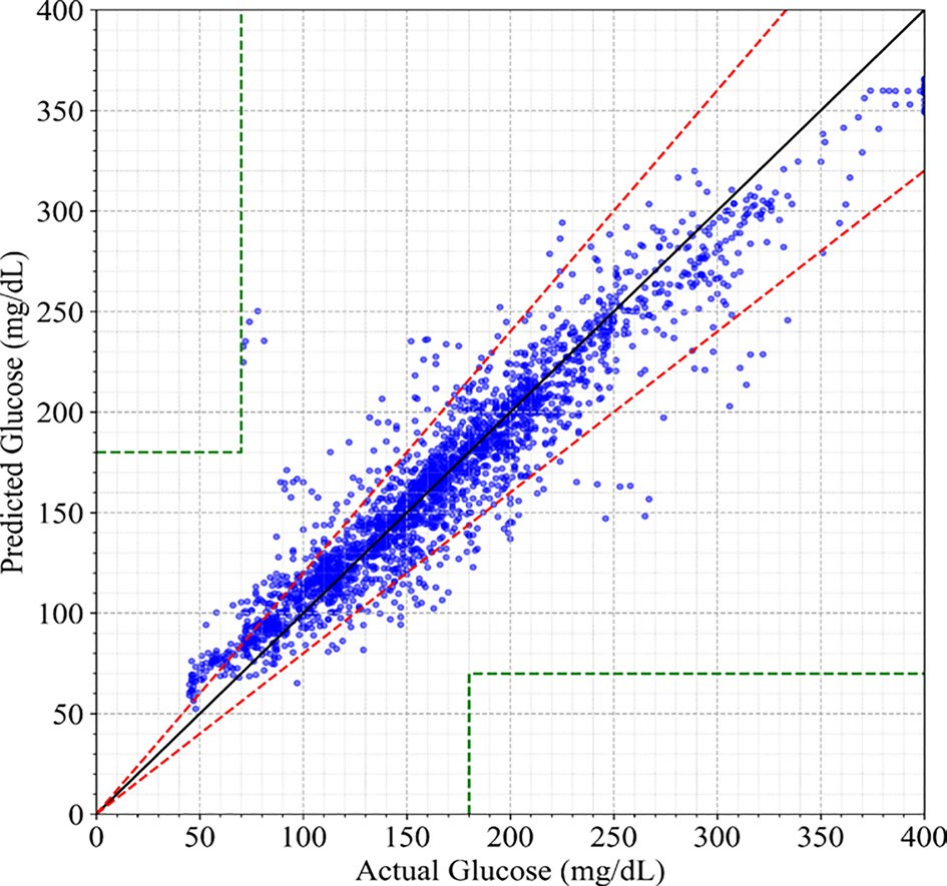
The clark grid error of the predicted values.

Furthermore, To assess the clinical relevance of the proposed forecasting model, the Clarke Error Grid (CEG) analysis is performed and visualized in Fig. 8. This reveals that 86.24% of the predictions fell within Zone A, indicating accurate and clinically acceptable readings, while 12.23% are in Zone B, representing benign errors with no significant impact on clinical decisions. Only 1.53% of the predictions falls into Zone C, which may lead to unnecessary treatment but does not pose critical risk. Notably, no predictions are found in Zones D or E, which are associated with dangerous or potentially life-threatening errors. These findings affirm the clinical utility and safety of the proposed model.

In addition to the CEG analysis, class-specific performance metrics are computed for hypoglycemia, normoglycemia, and hyperglycemia ranges. Table 7 presents the precision, recall, and F1-score for each class. The model demonstrates strong performance in detecting hypoglycemic events, achieving a precision of 0.776, a recall of 0.859, and an F1-score of 0.912, underscoring its clinical utility in managing low glucose episodes. For normoglycemia, while the recall is high (0.953), indicating that the majority of normoglycemic values are correctly identified, the lower precision (0.493) suggests some overlap with adjacent classes. Hyperglycemia detection showed a balanced performance with a precision of 0.703, recall of 0.903, and F1-score of 0.829. These results confirm that the model not only provides clinically safe predictions, as supported by the CEG, but also exhibits strong discriminative ability across glycemic states, particularly in critical hypoglycemic ranges.

**Table 7:** Precision, recall, and F1-score for each glycemic class.

Glycemic Class	Precision	Recall	F1-Score
Hypoglycemia (<70 mg/dL)	0.776	0.859	0.912
Normoglycemia (70–180 mg/dL)	0.493	0.953	0.760
Hyperglycemia (>180 mg/dL)	0.703	0.903	0.829

### Model explainability

To clinically interpret the proposed forecasting framework, explainable AI techniques are employed. Specifically, SHAP (SHapley Additive exPlanations) values are computed to assess the contribution of each engineered feature to model predictions. Table 8 summarizes the mean absolute SHAP values across the dataset. The average glucose over the past hour (avg) emerged as the most influential feature, followed by trend and time-of-day–related features. This ranking is consistent with the feature importance derived from the decision tree analysis (Table 4), thereby linking engineered feature relevance to the internal behavior of the RNN.

**Table 8:** Mean absolute SHAP values per feature.

Feature	Mean |SHAP| Value
avg	13.012
trend	5.009
day part	4.000
diff6	4.022
std	3.003
hour of day	3.002
day of week	2.003
diff1	1.626

**Fig. 9:**
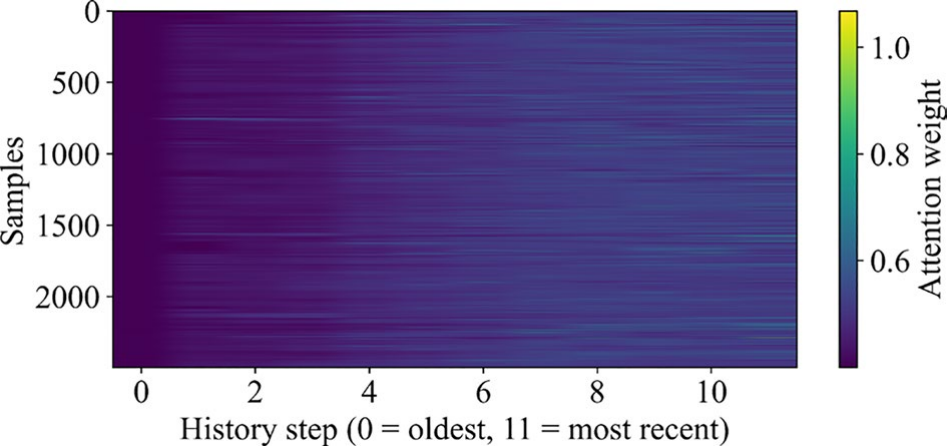
Attention weight heatmap across samples and historical time steps. More recent history steps received higher weights.

In parallel, the temporal focus of the RNN is investigated using an attention mechanism. The attention weight heatmap in Fig. 9 illustrates the relative importance assigned to historical glucose readings across all samples. The results indicate that more recent history steps (closer to the prediction point) received greater attention compared to older steps, which aligns with clinical expectations that near-term values exert stronger influence on short-term glucose forecasts.

By combining feature-level interpretability through SHAP with temporal interpretability via attention visualization, the proposed framework provides complementary insights into model behavior. This dual-perspective explainability improves transparency, builds clinical trust, and demonstrates that the model captures both engineered feature relevance and short-term temporal dependencies effectively.

### Comparison with similar studies

Although the proposed model can be considered as a simple and light weight one, it outperforms other architectures either in accuracy or complexity as summarized in Table 2.

Regarding^21^, it demonstrates a marginally lower mean RMSE compared to the proposed work. Nevertheless, the model exhibited similar performance with specific patients, such as 563, 570, and 591, while surpassing the performance of^21^ with patient 575, achieving an RMSE of 21.52 compared to their RMSE of 22.7. Moreover, despite methodology presented in^9^ being limited to just six patients compared to the comprehensive work on all 12 patients in this study, the RMSE values are remarkably the same. Notably, the model outperforms theirs in the case of patients 567 and 584, with RMSE values of 20.57 and 21.57, respectively, as opposed to their RMSE values of 22.76 and 22.22 for the same patients. while in the context of^8^, their results show the same performance level to the proposed model, despite employing an ensemble model comprising LSTM, bidirectional LSTM, and a linear model, which represents a significantly more complex architecture than ours. Same with^14^ which introduced an intricate combined model of LSTM, WaveNet, and GRU, however, the model demonstrates superior performance.

Beyond achieving competitive forecasting accuracy, the clinical significance of the proposed model lies in its ability to anticipate critical glucose fluctuations with sufficient lead time for intervention. A 30-minute prediction horizon enables patients and caregivers to proactively adjust insulin dosages, meal timing, or activity levels, potentially preventing life-threatening hypoglycemic or hyperglycemic events.

### Considerations and Limitations

This study was conducted using data from 12 patients extracted from a high-resolution CGM dataset. While the dataset provided rich temporal and contextual information, the relatively small sample size presents limitations in terms of generalizability. The chosen patients may not fully represent the diversity of glucose dynamics found in broader diabetic populations, including lifestyle, comorbidities, and diabetes management strategies. As a result, the current findings—though valid within this controlled cohort—may not fully capture the variability and complexity observed in real-world settings. There is a potential risk of sampling bias, where the model may inadvertently perform better for the specific data distribution of this group. Future work should focus on validating the framework using larger and more heterogeneous populations, ideally through collaborations with clinical centers or real-world deployments.

### Real-world application

The proposed glucose forecasting framework is designed with a clear focus on real-world deployment, particularly as part of a cloud-based infrastructure integrated with CGM devices.

**Fig. 10:**
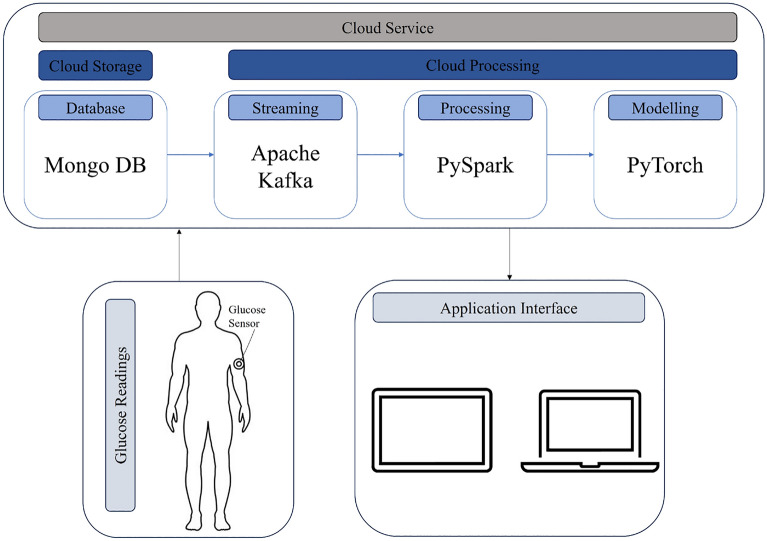
System integration diagram illustrating glucose sensing, cloud storage/processing, and application interface.

The system architecture is illustrated in Fig.10, where glucose readings are collected via wearable sensors and streamed to a cloud service. The cloud infrastructure incorporates MongoDB for storage, Apache Kafka for streaming, Spark for data processing, and PyTorch for model training and inference. The predicted outputs are then delivered through an application interface accessible via both mobile and web platforms. This end-to-end design ensures real-time processing and user feedback, supporting early alerts for hypoglycemia and hyperglycemia. By integrating the forecasting model with scalable cloud services and patient-facing applications, the framework demonstrates feasibility for real-world deployment and accessibility for both clinical and personal diabetes management.

### Timing analysis

To further evaluate the computational efficiency of the proposed framework, a comparison was conducted with alternative time-series layers including LSTM, GRU, and a simple 1D CNN baseline. This analysis was performed to empirically demonstrate that the RNN is the most lightweight architecture for this application. All experiments were run on a system equipped with an Intel(R) Core(TM) i7-7700HQ CPU @ 2.80GHz, 16 GB RAM, SSD NVMe storage, and an NVIDIA GeForce GTX 1050 GPU. Table 9 summarizes the training time, inference latency, and peak memory usage for each model when trained on a single patient dataset.

**Table 9:** Runtime and memory profiling of RNN vs. alternative architectures.

Model	Training Time (s)	Inference Time (s)	Peak GPU Memory (MB)
RNN	106.48	0.0078	414
LSTM	152.22	0.0086	1082
GRU	135.16	0.0073	840
CNN	167.36	0.0076	410

The results confirm that the RNN requires significantly less training time and GPU memory compared to LSTM and GRU, while maintaining comparable inference latency. Although the CNN demonstrated similar GPU memory usage to the RNN, it exhibited a longer training time. These findings support the conclusion that the RNN is a computationally efficient and lightweight model, making it well-suited for real-time glucose forecasting.

## Conclusion

Diabetes mellitus is a chronic illness that necessitates routinely checking blood glucose levels in the circulatory system. For the affected person, failing to maintain glucose levels within a certain range can have serious implications. As a result, a large number of studies in the corpus of extant research focus on the forecasting of BG levels. In this context, the creation of a blood glucose level forecasting model is the focus of our research activities, with the main objective being the accurate prediction of glucose levels with a lead time of 30 minutes. The primary areas of expertise are the application of cutting-edge methods for managing missing data and carrying out feature engineering. The overarching objective is to contribute to the prevention of diabetes by providing patients with sophisticated insights about their glucose trends. RNN model is applied to forecast blood glucose levels with a lead time of 30 minutes. The model’s performance was assessed using the RMSE metric, which is a crucial indicator of predictive accuracy. The RMSE values ranged between 15.96 and 21.57, with a margin of error falling within the range of 0.03 to 0.45. The model showed strong predictive performance for certain individuals but struggled to accurately capture the intricacies of glucose fluctuations in others. Nevertheless, this study marks a significant advancement in providing patients with valuable insights into their glucose trends, aligning with the broader objective of diabetes prevention.

## Data Availability

The OhioT1DM dataset used in this study is not publicly available. Access to this dataset can be obtained by researchers through a Data Use Agreement (DUA) with Ohio University. The dataset is detailed in the publication Marling, C. et al., OhioT1DM Dataset for Blood Glucose Level Prediction, available at https://pubmed.ncbi.nlm.nih.gov/33584164/, DOI: 10.2196/16662.
